# Cancer trends and risk factors in China over the past 30 years (1990-2019)

**DOI:** 10.7150/jca.83162

**Published:** 2023-06-26

**Authors:** Weiwei Wu, Ruochen Zhang, Yiming Jin, Yan Lu, Zhonglei Lu, Tao Li, Liefu Ye, Le Lin, Yongbao Wei

**Affiliations:** 1 Department of Neurology, Union Hospital, Fujian Medical University, Fuzhou, China.; 2 Shengli Clinical Medical College of Fujian Medical University, Fuzhou, 350001, China.; 3 Department of Urology, Fujian Provincial Hospital, Fuzhou, 350001, Fujian, China.; 4 Department of Urology, Juntendo University School of Medicine, Tokyo, Japan.; 5 College of Biological Science and Engineering, Fuzhou University, Fuzhou 350108, China.

**Keywords:** Cancer trends, risk factor, China, cancer mortality, cancer control, GDP

## Abstract

**Objective:** We retrospectively studied cancer mortality and incidence in China from 1990 to 2019, investigated the cancer trends and risk factors, and analyzed the effects of Gross Domestic Product (GDP) on cancer mortality and incidence.

**Methods:** Data was obtained in "Our world in data" in October 2022 to explore mortality rates of different cancers and their trends and the roles of cancer risk factors, including GDP, air pollution, etc.

**Results:** Over the past 30 years, cancer had been China's second leading cause of death. Tracheal, bronchial, and lung cancers, with an annual growth rate of 6.5%, were the most frequently diagnosed cancers. The burden of different cancers changed as the mortality rate of cancer changed. The age-standardized cancer mortality rate had decreased by 19.0%; cancer deaths in all age groups had increased. While the number of cancer deaths in the elderly aged ≥70 did not increase distinctively, its percentage increased by 52.1% and 1.7% annually. The percentage of patients with new-onset cancer increased by 240% and 8.6% annually. For every USD 1,000 increase in GDP, cancer deaths decreased by 2.3/100,000. Tobacco, meat, and alcohol consumption and BMI had increased and were not conducive to the future control of cancer.

**Conclusions:** We summarized the incidence and mortality of major cancers and their trends in China over the past 30 years and analyzed the effects of GDP and the roles of cancer risk factors. Overall GDP growth and effective control of air pollution reduced cancer mortality, while population aging, smoking, alcohol consumption, BMI increasing, and meat consumption brought challenges for cancer control.

## Introduction

Cancer is one of the leading causes of death in China 1. As the population ages, changes in cancer risk factors, particularly diet, obesity, diabetes, and air pollution, have led to a shift in the approaches of the Chinese government to cancer control. Although many achievements have been made, challenges have remained 2. Lung cancer is currently the leading cause of cancer-related deaths in China. Although the burden of upper gastrointestinal cancer is decreasing, the burden of colorectal, prostate, and breast cancers is increasing annually 2. In terms of gender differences, gastrointestinal cancers, including gastric, colorectal, liver, and esophageal cancers, cause a large cancer burden in both men and women, while breast cancer is the most common type of cancer in women 3. Lung cancer consistently had the highest mortality and morbidity among men, while prostate cancer had the second highest age-standardized incidence rate among men, increasing by 112% from 1990 to 2017 4. The cancer spectrum in China was changing; in addition to the high incidence and burden of liver, stomach, esophagus, and cervical cancers, the incidence and burden of other cancers (lung, breast, colorectal, and prostate) were also rapidly increasing. China's cancer spectrum shifted from developing to developed countries 5. However, these dynamic change data reports have been relatively limited over the last 30 years and deserve further exploration.

Population-based cancer registries have been operational in China for approximately 60 years; they are a crucial component of China's health priorities, which are also responsible for progress tracking in cancer control 6. Healthy China 2030 paints a bright vision for the future of cancer control in China 7. However, reducing the cancer burden in China requires cross-sectoral collaborative and coordinated efforts by the government, public health organizations, and individuals in primary and secondary prevention, and the situation does not appear to be optimistic 2,8. The purpose of this study was to retrospectively study the trends in cancer incidence and mortality in China over the past 30 years (from 1990 to 2019) and to examine the recognized risk factors for cancer in cancer control, including smoking, alcohol consumption, meat consumption, air pollution, BMI, and gross domestic product (GDP), to provide data support for strategies to achieve China's 2030 Cancer Control Plan.

## Method

We obtained permission to attain data on cancer and lifestyle habits in China from "Our world in data" (https://ourworldindata.org/) in October 2022 9. This database is intended to address major issues occurring around the world, including major public concerns about cardiovascular diseases, cancer, air pollution, water pollution, dietary hygiene, and the recent coronavirus disease 2019 (COVID-19) pandemic 10. This database is fully accessible, and the data owners confirmed that we could use, reproduce, and distribute it in any medium as long as the source and its authors were cited accordingly. Therefore, we collected data on all mortality rates in annual units in China from 1990 to 2019 and plotted the trend curves of various causes of death over time. We then focused on the mortality rates and trends of different cancer types to explore age-related mortality rates for different cancers and their trend curves, analyze age-based cancer incidence and its trends, and explore the cancer burden in China over this period. Comparing the percentage of cancer deaths over time with a baseline of 1990, the cancer death rate included both unstandardized and age-standardized cancer death rates. We explored the proportion of non-genetic factors contributing to cancer death in different cancer types, including a wide range of non-genetic external environmental factors such as exposure to carcinogens, GDP, environment, smoking, alcohol consumption, meat consumption, and BMI, which are related to cancer occurrence and death, to provide data reference for reducing cancer deaths in China. All data are presented as numbers and percentages.

## Results

### Cancer mortality and its trends

**Figure 1:**
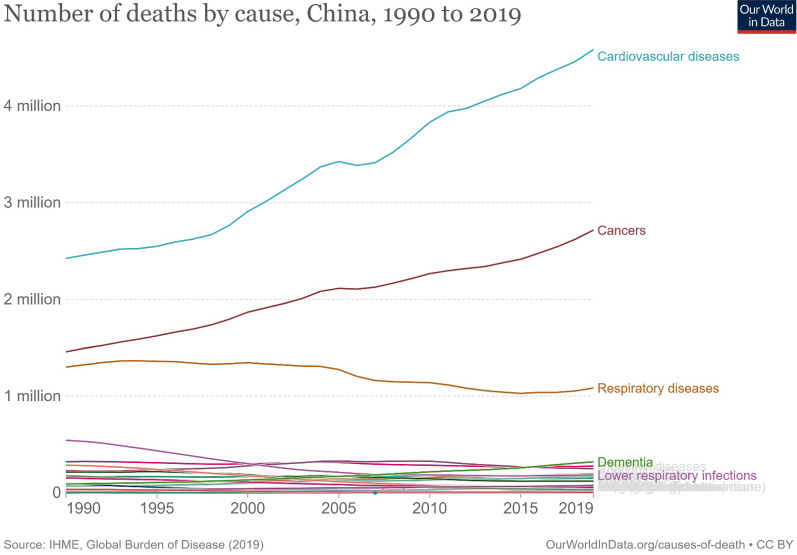
The number and trends of deaths by causes in China from 1990 to 2019.

**Figure 2:**
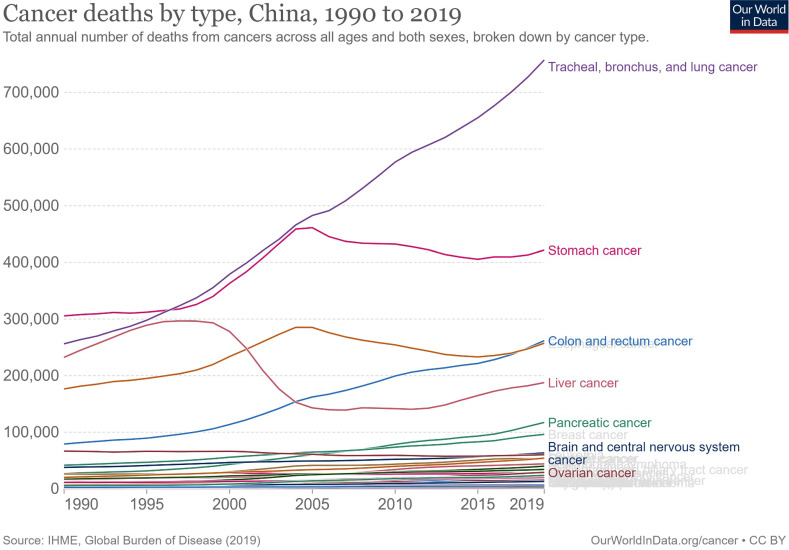
Cancer deaths by types in China from 1990 to 2019.

Cancer deaths in China increased from 1.46 million to 2.72 million in 2019, second only to cardiovascular diseases at 4.58 million and 1.63 million more than the third-ranked respiratory diseases (Figure 1). In 1990, the top five cancer deaths were stomach cancer (n = 305,467); tracheal, bronchial, and lung cancer (n = 256,326); liver cancer (n = 232,449); esophageal cancer (n = 176,602); and colon and rectum cancer (n = 79,322). By 2019, the number of cancer deaths had changed, ranking first as tracheal, bronchial, and lung cancers (n = 757,171), with an increase of 195.4% and 6.5% annually. This was followed by stomach cancer (n = 421,539, an increase of 37.9% in total and 1.3% annually), colon and rectal cancer (n = 261,777, an increase of 230.0% in total and 7.7% annually), esophageal cancer (n = 257,316, an increase of 45.7% and 1.5% annually), and liver cancer (n = 187,700, a decrease of 19.3% in total and 0.6% annually). The subsequent fastest-growing cancers were panic cancer (ranked 6^th^ in 2019) and breast cancer (ranked 7^th^ in 2019), which increased by 333.1% and 130.4%, respectively, in 30 years, with respective annual growth rates of 11.1% and 4.3% (Figure 2, Supplemental Figure 1).

**Figure 3:**
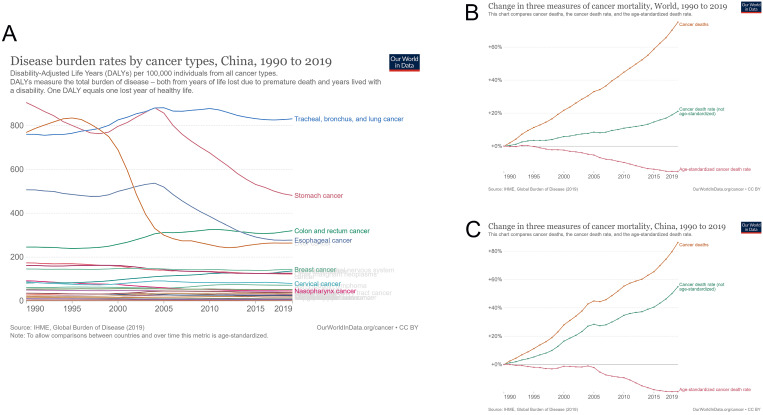
Cancer burden in China from 1990 to 2019. (A) Disease burden rates by cancer types; (B) changes in three measures of cancer mortality in the world from 1990 to 2019; (C) changes in three measures of cancer mortality in China from 1990 to 2019.

The cancer burden in China has also changed over the past 30 years, and the trend has changed according to cancer mortality. The maximum cancer burden was caused by gastric cancer in 1990, which changed to tracheal, bronchial, and lung cancers in 2019. Among the top five cancers, both esophageal and liver cancer burdens decreased, while colon and rectal cancer burdens steadily increased yearly (Figure 3A). As a result, the cancer mortality rate in China has increased yearly since 1990. By 2019, the cancer mortality rate in China had increased by 86.2%, and the age-unstandardized cancer mortality rate had increased by 54.9%. In contrast, the age-standardized cancer mortality rate dropped significantly (19.0%) in 2019, compared with 1990. Compared with the world cancer mortality rate, China's cancer mortality rate increased more over the past 30 years (world cancer mortality rate increased by 75.1%); however, China's age-standardized cancer mortality rate decreased more evidently (world age-standardized cancer mortality rate decreased by 15.2%) (Figure 3B and 3C).

### Age-related cancer mortality and trends

**Figure 4:**
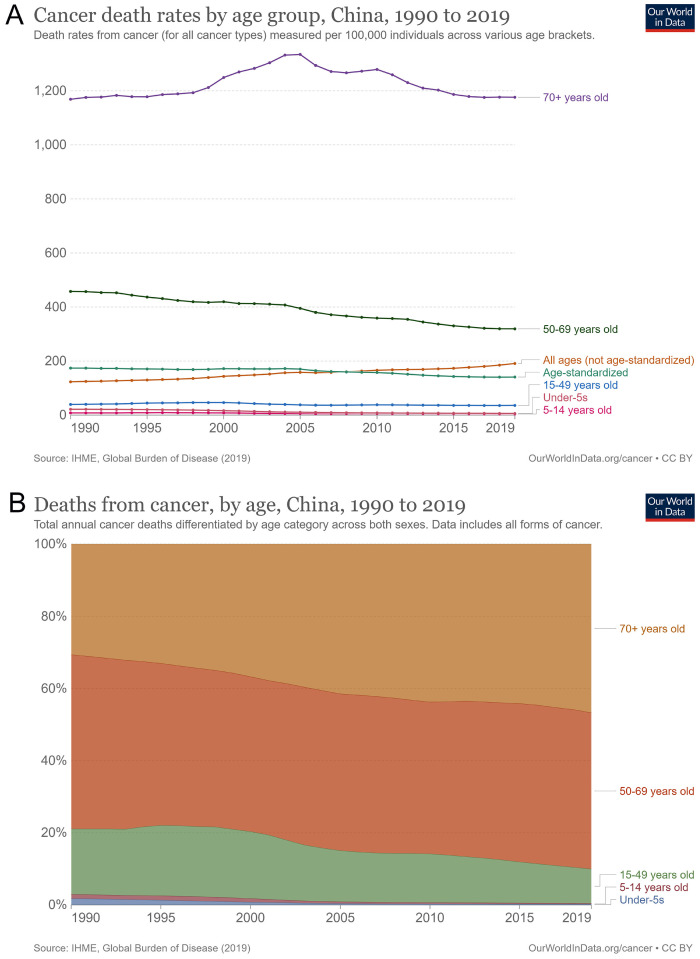
Cancer death by age in China from 1990 to 2019. (A) Cancer death rates by age groups; (B) percentage of cancer deaths among age groups.

Cancer mortality rates in different age groups have changed annually. The number of cancer deaths in all age groups increased from 123.3/100,000 in 1990 to 190.9/100,000 in 2019, an increase of 54.9% in total and 1.8% annually. However, the rate of age-standardized cancer mortality showed a downward trend, a decrease of 19.0% in total and 0.6% annually. Cancer deaths in the elderly aged ≥ 70 years first increased (1334.5/100,000 in 2005) and then decreased, and the increase was not apparent until 2019 (Figure 4A). As a result, the percentage of cancer deaths in the elderly aged ≥ 70 years increased from 30.7% in 1990 to 46.7% in 2019, an increase of 52.1% in total and 1.7% annually. Cancer deaths in people aged 50-69 years had decreased from 457.5/100,000 to 319.1/100,000, a decrease of 30.3% in total and 1.0% annually. The percentage of people aged 50-69 also dropped from 18.1% in 1990 to 9.5%, a decrease of 47.5% in total and 1.6% annually. The numbers of other age groups did not increase; however, their percentages decreased across all age groups (Figure 4B).

### Prevalence of age-related cancer incidence

**Figure 5:**
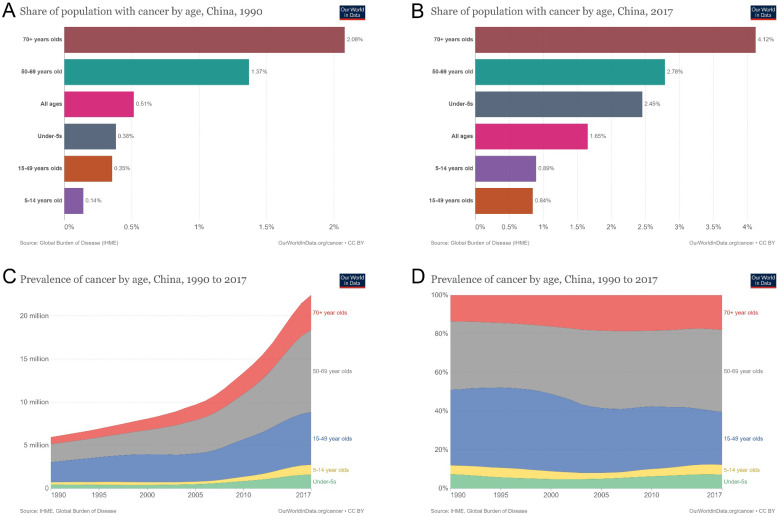
Prevalence of age-related cancer incidence in China from 1990 to 2017. (A) population with cancer by age in China in 1990; (B) population with cancer by age in China in 2017; (C) prevalence of cancer by age in China from 1990 to 2017; (D) percentages of cancer prevalence by age in China from 1990 to 2019.

Cancer incidence in different age groups increased from 0.5% of the population in 1990 to 1.7% in 2017. The percentage of patients with new-onset cancer in the population increased by 240% and 8.6% annually (Figures 5A and 5B). The number of patients with new-onset cancer aged ≥ 70 years was 0.8 million in 1990 and 4.1 million in 2017, an increase of 412.5% ​​in total and 14.7% annually. The number of patients aged 50-69 years increased from 2.1 to 9.53 million, an increase of 353.8% and 12.6% annually. The number of patients aged 15-49 increased from 2.3 million to 6.1 million, 165.2% in total and 5.9% annually (Figure 5C). Among the proportions of patients of different ages, the proportion of patients with new-onset cancer aged ≥ 70 years increased from 13.6% to 18.1%, a growth rate of 33.1% in total and 1.2% annually. The proportion of patients with new-onset cancer aged 50-69 increased from 35.5% to 42.5%, a growth rate of 19.7% in total and 0.7% annually. For patients with new-onset cancer aged 15-49 years, the percentage decreased from 39.1% to 27.2%, a decline rate of 30.4% in total and 1.1% annually (Figure 5D).

### Cancer death related to economic and non-genetic risk factors

**Figure 6:**
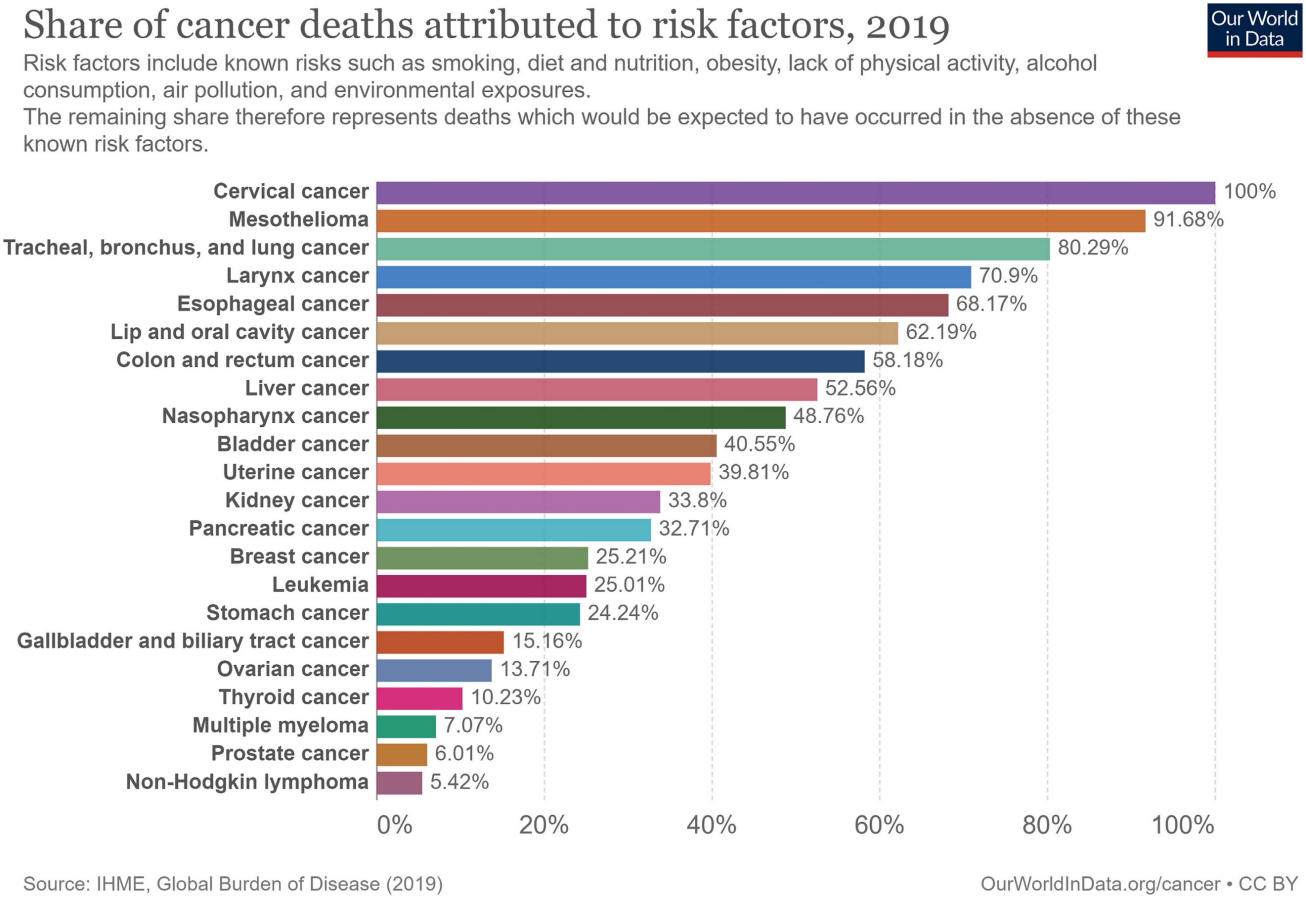
Cancer death attributed to non-genetic risk factors in 2019.

We found that the contribution of economic and non-genetic risk factors to cancer mortality differed, and many cancers appeared preventable and controllable. The non-genetic risk factors contributing to cervical cancer mortality risk were set as 100% (baseline); mortality risks of other cancers were lower than that of cervical cancer. The top five cancer-related deaths contributed by non-genetic risk factors were cervical cancer (100%), followed by mesothelioma (91.7%); tracheal, bronchial, and lung cancers (80.3%); laryngeal cancer (70.9%); esophageal cancer (68.2%); and lip and oral cavity cancer (62.2%). Among the other 6^th^ to 22^nd^ cancers, cancer deaths attributed to non-genetic risk factors were other digestive system cancers, urinary and reproductive system cancers, and hematological malignancies (Figure 6).

**Figure 7:**
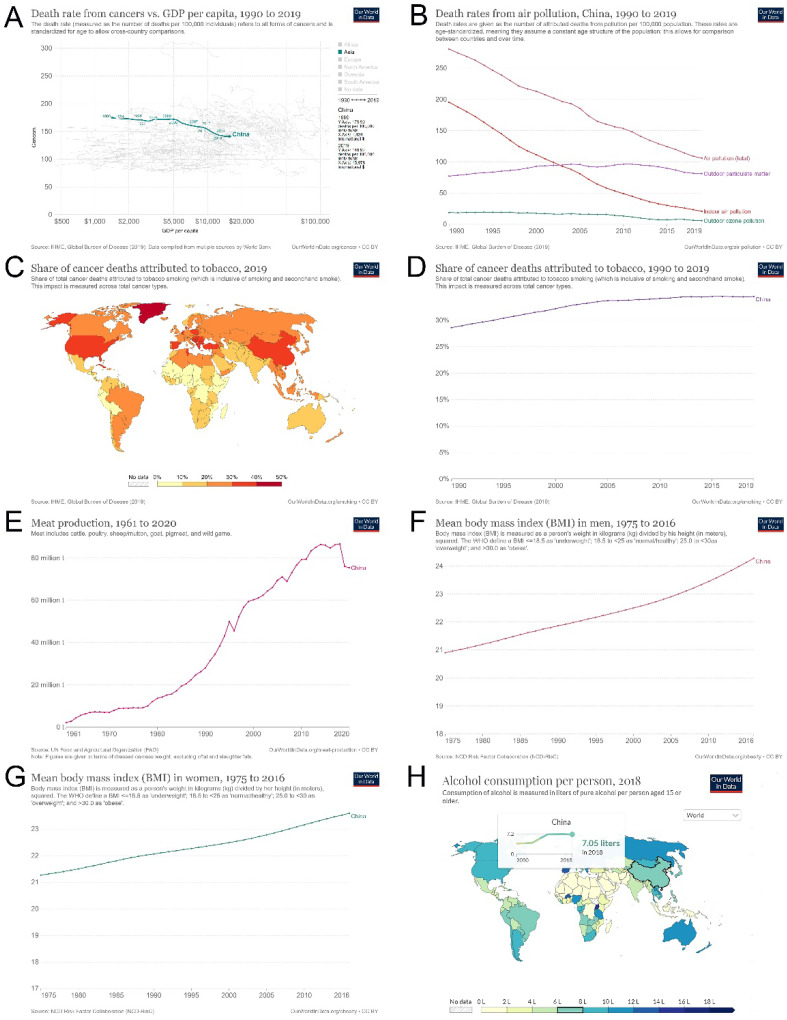
Cancer death attributed to economic and non-genetic risk factors in China since 1990. (A) GDP per capita and cancer death rates in China from 1990 to 2019; (B) air pollution and deaths in China from 1990 to 2019; (C) cancer deaths attributed to tobacco in the whole world in 2019; (D) cancer deaths attributed to tobacco in China from 1990 to 2019; (E) meat consumption in China from 1961 to 2020; (F) Mean body mass index in men in China from 1975 to 2016; (G) Mean body mass index in women in China from 1975 to 2016; (H) Alcohol consumption per person in China from 2000 to 2018.

We found that China's economic development over the past 30 years has helped to reduce cancer mortality. With the annual increase in GDP, the cancer death rate decreased from 173.9/100,000 in 1990 to 140.9/100,000 in 2019. For every $1,000 increase in GDP, cancer deaths decreased by 2.3/100,000 (Figure 7A). Similarly, effectively controlling air pollution may reduce the mortality rate to a certain extent (Figure 7B). However, tobacco consumption in China remained high (28.62% in 1990 and 34.51% in 2019), was at a relatively high level worldwide, and tended to increase yearly, which is not conducive to cancer control (Figure 7C and D). In addition, the overall trend of unfavorable diet habits, such as meat consumption, increased evidently over the years (Figure 7E). The BMI of both men and women also increased annually (men, 21.9 kg/m^2^ in 1990 and 24.3 kg/m^2^ in 2016 (Figure 7F); women, 22.1 kg/m^2^ in 1990 and 23.6 kg/m^2^ in 2016 (Figure 7G). Furthermore, the overall trend of alcohol consumption increased over the past years, from 3.81 liters per person in 2000 to 7.05 liters per person in 2018 (Figure 7H). These unfavorable risk factors may contribute to the current increase in new-onset and cancer deaths.

## Discussion

We found that tracheal, bronchial, and lung cancer surpassed gastric cancer as the leading cause of cancer mortality over the past 30 years, and its mortality rate increased at an annual rate of 6.5%. Tobacco exposure is a significant factor driving current trends in lung cancer 11. Smoking rates have remained high in recent decades 2. We found that the cancer mortality rate caused by smoking increased and has been at a high proportion worldwide. Additionally, a meta-analysis found that non-smokers with substantial exposure to secondhand smoke had a significantly increased overall cancer risk compared to those without such exposure (odds ratio (OR) of 1.163 (95% CI 1.058-1.279)); secondhand smoke significantly increased the risk of cancer in women (OR 1.253, 95% CI 1.142-1.374), including the risk of lung cancer (OR 1.245, 95% CI 1.026-1.511); thus, it was not only necessary to reduce active smoking but also to limit exposure to secondhand smoke 12. China has set strict targets for tobacco control, and Healthy China 2030 targets to reduce smoking prevalence by 20% 7. Therefore, smoking-related cancer morbidity and mortality may vary in the future. The overall trend of total lung cancer mortality in China is expected to decline over the next decade (2020-2030); however, the death rate is expected to continue to increase in the context of the aging population and high smoking rates in China 13. The implementation of tobacco control programs faces multiple challenges. In addition to the enormous economic benefits of tobacco and other socioeconomic factors, it is difficult to impose a sudden ban on tobacco, considering individuals with tobacco abuse 14. No significant difference in mean smoking cessation rates was observed between patients with/without cancer, regardless of whether smokers currently had cancer, were cancer survivors, or had a history of smoking-related cancers compared to those without a history of cancer 15.

The mortality and burden of digestive cancers vary widely. Cancer mortality rates of stomach, esophagus, and colorectal cancers increased slightly (annual growth rate, 1.3%-7.7%), whereas pancreatic cancer mortality rates increased rapidly (annual growth rate, 11.1%). Although the age-standardized burden of esophageal cancer has declined, new cases and deaths are predicted to continually increase 16. Given the pattern of hepatitis B virus infection, China has long contributed to more than half of the global liver cancer burden in the past; however, a decline in the incidence of liver cancer across the country was observed recently, which may be related to the implementation of primary and secondary prevention interventions; it was predicted that liver cancer incidence in China may drop by 50% in 2050 17. The overall mortality trend of digestive system cancers (liver, stomach, and esophagus) in China is expected to decline over the next decade (2020-2030); however, the related deaths are expected to surge in the context of continued aging and high smoking rates 13. Age-standardized rates of pancreatic cancer incidence and mortality increased from 1990 to 2019 and were higher in men than women; in particular, its incidence and mortality in the > 25 age group increased yearly 18. The number of cases and deaths of pancreatic cancer is estimated to increase to 218,790 and 222,970 in 2030, respectively, an approximately 2-fold increase 19.

We found that the current proportion of reproductive system cancers was lower than that of lung and digestive system cancers; however, their incidence increased yearly; this may change the trend of cancer mortality and burden in the future. In men, the age-standardized incidence of prostate cancer in China increased by 12.6% from 2000 to 2011 (the highest rate of increase in cancers) 4. In 2020, prostate cancer was the second most common malignant tumor in men worldwide, accounting for 14.1% of all male cancer incidence 20. In women, the age-standardized incidence rates of breast, cervical, and ovarian cancers have also increased sharply in China 2. Female breast cancer has surpassed lung cancer as the most commonly diagnosed cancer worldwide and ranks fifth in cancer deaths 20. The impact of reproductive system cancer will increase sharply in the future 20,21. Regarding risk factors, both smoking and secondhand smoke significantly increase the risk of breast cancer 12. Tobacco control is expected to reduce the incidence and mortality of breast cancer to a certain extent.

Age is a critical factor in the incidence and mortality of cancer. Cancer mortality in all age groups in China has increased by 54.9% over the past 30 years. However, the age-standardized cancer mortality rate has decreased by 19.0% and 0.6% annually. Aging has increased cancer morbidity and mortality, and although aging did not increase the number of age-standardized cancer deaths, the percentage of cancer mortality across all age groups showed a noticeable increase. For example, the number of cancer deaths in the elderly aged ≥ 70 did not increase significantly; however, its percentage increased by 52.1% and 1.7% annually. By the end of 2022, there will be approximately 4.82 million and 2.37 million new cancer cases and 3.21 million and 640,000 cancer deaths in China and the United States, respectively; the increase in the number of the adult and aging population may be the main determinants of the increase in cancer deaths; therefore, active response to population aging may help reduce the cancer burden in China 21.

Communicating diseases are not conducive to cancer control. The global pandemic, caused by a novel coronavirus called “severe acute respiratory syndrome coronavirus 2,” continues. Although the cause of death from COVID-19 is multiple organ dysfunction syndrome, patients with cancer are more susceptible to infection than those without cancer and seem to have a poorer prognosis due to their systemic immunosuppression status 22. Chemotherapy within four weeks before the onset of COVID-19 symptoms is a risk factor for death during hospitalization 23. A systematic review and meta-analysis found that patients with cancer were more susceptible to COVID-19 infection, and cancer increased the mortality rate of patients with COVID-19, among which patients with lung cancer had higher mortality rates than those without lung cancer 24. Although vaccination against COVID-19 has helped to reduce its severe morbidity and mortality, there was considerable controversy regarding the vaccination for patients with cancer, including vaccine efficacy, the degree of humoral and cellular immune responses in these patients, and the incidence of vaccine-related adverse events 25. This controversy has increased the difficulty of cancer epidemic prevention and control.

GDP has been associated with fewer cancer deaths. We found that for every $1,000 increase in China's GDP, cancer deaths decreased by 2.3/100,000. Although countries with higher GDP per capita have higher cancer incidence rates, the mortality rates have fallen substantially 26. In countries with declining tobacco use, bladder cancer mortality was inversely related to per capita GDP 27. GDP was also positively associated with testicular cancer incidence but negatively associated with testicular cancer mortality 28. In addition, total health expenditure/GDP was related to a decrease in the ratio of morbidity and mortality of cancers such as stomach and lung cancers, suggesting that the total health expenditure was directly proportional to GDP and inversely proportional to the cancer mortality rate 29,30. The increase in GDP has also contributed to the implementation of cancer research in China. Cancer research publications in China have proliferated over the past decade and surpassed the United States in 2018. Compared with other Asian countries, China's output was roughly proportional to its wealth 31, which may also contribute to a reduction in the mortality rate of patients with cancer. In addition, China's commitment to environmental pollution, such as effectively controlling air pollution, may also reduce the risk of cancer-related deaths 32.

Poor lifestyle habits increase the difficulty of cancer control. We found that China's meat and alcohol consumption showed an overall increasing trend, resulting in increased BMI every year. The study found that red meat consumption was associated with an increased risk of mortality from multiple hematological and solid cancers; an increase of 100 g of red meat per day was associated with an 11%-51% increase in the risk of multiple cancers, while an increase of 50 g per day of processed meat consumption was associated with an 8%-72% increase in cancers 33. From 1990 to 2019, an increase in colorectal cancer mortality due to a high intake of processed meat was observed for both sexes in China; thus, gradually reducing the intake of processed meat may be an effective way to reduce colorectal cancer mortality 34. We found that the current cancer risk corresponding to Chinese men's and women's BMI was within the controllable range. A study found that people with a BMI of approximately 22-25 had the lowest risk of cancer in the overall sample; however, a 1-unit increase in BMI was associated with a 5% increased risk of death in overweight participants (BMI 25.0-29.9) and 9% in obese participants (BMI ≥ 30.0). In comparison, a 34% and 14% lower risk was found in underweight (BMI < 18.5) and low-normal-weight participants, respectively 35. With the development of the social economy, the BMI burden on cancer prevention and treatment will gradually increase if it is not controlled. The prevalence and number of individuals overweight and obese and associated medical costs are predicted to burden China's healthcare system by 2030. China has undertaken considerable to address obesity, including implementing related national policies and planning; however, these measures appear insufficient to control the obesity epidemic 36. Globally, an estimated 741,300 or 4.1% of all new cancer cases in 2020 could be attributed to alcohol consumption, particularly alcohol-induced cancers such as the esophagus, liver, and breast; thus, measures are needed to increase awareness of alcohol use-related cancer risks and reduce overall alcohol consumption to prevent alcohol-induced cancer burden 37.

In China, both opportunities and challenges exist for cancer prevention and control. The continuous growth of China's GDP, effective control of environmental pollution, and popularization of clean energy will help reduce the incidence and mortality of cancer. In addition, China's progress and capabilities in cancer drug research and development and cancer clinical research have contributed to the management of patients with cancer. Furthermore, several recent health policies proposed by China have pointed to China's nutrition policy improvement over the next decade 38. However, the number of cancer clinical trials with unique epidemiological characteristics in the Chinese population is small, and the geographical distribution is uneven. Therefore, it is necessary to address uniquely related and rare cancers in the Chinese population and the fairness, efficiency, and sustainability of cancer drug development 39. Notably, the low participation rate in China's cancer screening, unevenly distributed medical resources, insufficient funds, and insufficient screening quality have caused difficulty in cancer control. In addition to addressing these issues, it is necessary to increase personnel training, optimize the definition of high-risk groups, and integrate new technologies into cancer screening programs in the future 40. Furthermore, strengthening the 1^st^ and 2^nd^ levels of cancer prevention, particularly for those living in rural areas and vulnerable groups, would contribute to the overall control of cancer in China 1. However, the frequent occurrence of communicable diseases worldwide, including in China, is not conducive to cancer screening and control. For example, cancer screening programs have been interrupted due to the onset of COVID-19, which has caused a delay in the diagnosis and, as a result, increased the mortality rate owing to cancer deaths that would have been avoidable in a pre-COVID era. Thus, urgent policy intervention is needed to address the backlog of routine diagnostic services and minimize the detrimental impact of the COVID-19 pandemic on patients with cancer 41.

This study had some limitations. Firstly, we did not re-analyze the data from "Our world in data"; thus, the results of this study were presented in descriptive form. Secondly, we gave the relative risk trends but did not analyze their correlations with cancer treads, morbidity, and mortality. Furthermore, this study did not include and analyze other cancer risks, such as the increasing population, the positive impact of medical technology advancements (cancer drug innovations, advances in surgical technology, etc.), and the effect of medical insurance policy on cancer incidence and death. In addition, China has a vast territory, and different regional food cultures differ significantly. Therefore, we did not give information about cancers with high incidence in different regions and their detailed change trend of cancer in different areas of China.

## Conclusions

We summarized the developmental trends of morbidity and mortality of major cancers in China over the past 30 years and calculated their annual growth rates. Lung cancer has the highest morbidity and mortality rate. In addition, the mortality rates of pancreatic and colorectal cancer among digestive system cancers and prostate and breast cancers in the reproductive system show a rapidly increasing trend and need strengthening. We also analyzed the effects of GDP and changes in cancer risk factors on cancer incidence and mortality. Overall GDP growth and effective control of air pollution reduced cancer mortality, which, however, continued to increase owing to population aging, smoking, alcohol consumption, BMI, and meat consumption; these factors are adversely affecting cancer control. Hence, it is necessary to alert and educate society regarding the need for control.

## Supplementary Material

Supplementary figure.
