# Supplementary material for: Cancer trends and risk factors in China over the past 30 years (1990-2019)
Source: J Cancer. 2023 Jun 26;14(10):1935–45. doi: 10.7150/jca.83162 (PMC10355210; doi:10.7150/jca.83162)
Supplement: Supplementary file 1 — Supplementary figure. [file jcav14p1935s1.pdf]

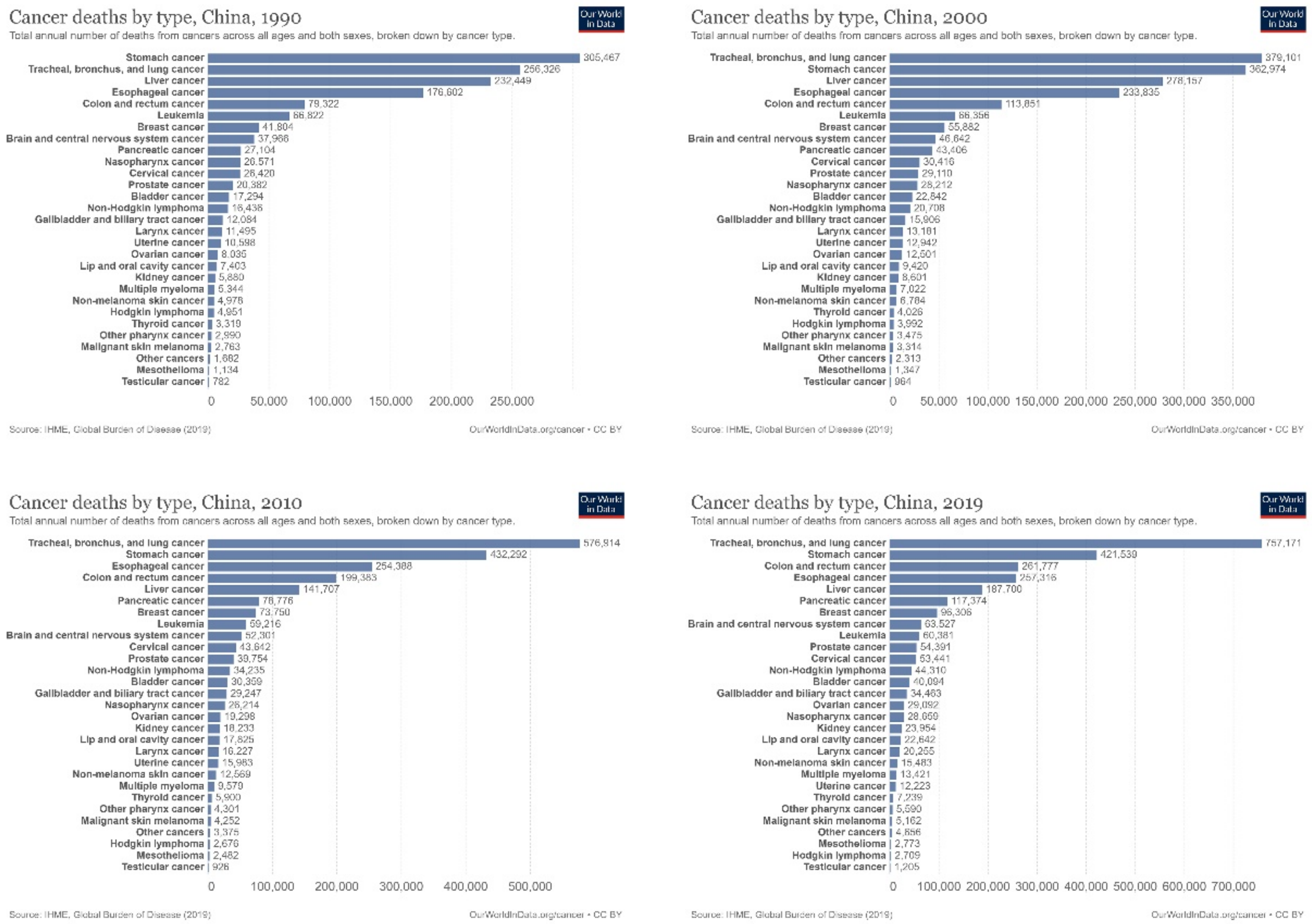

Supplement Figure 1. Cancer mortality and its trends.
